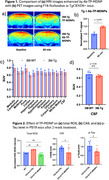# Targeting Neuroinflammation by Bioreactive Nanoparticles for Early Detection and Intervention of Alzheimer’s Disease

**DOI:** 10.1002/alz70859_104173

**Published:** 2025-12-25

**Authors:** Liting Wang, Elliya Park, Chunsheng He, Azhar Z. Abbasi, Raveen Christian Joy Rajakumar, Shudi Huang, Paul E Fraser, Jeffrey T. Henderson, Xiao Yu Wu

**Affiliations:** ^1^ University of Toronto, Toronto, ON Canada

## Abstract

**Background:**

Neuroinflammation plays a causal role in neurodegenerative Alzheimer’s disease (AD); it occurs long before clinical onset of AD.^1^ Therefore, early detection of neuroinflammation is critical for early intervention before the irreversible neurodegeneration happens. To address this pressing need, our group has developed multifunctional bioreactive nanoparticles, consisting of blood‐brain barrier‐penetrating terpolymer and MnO_2_ nanoparticles and conjugated anti‐Ab antibody (Ab‐TP‐MDNP). The system reduced oxidative stress and produced oxygen and paramagnetic Mn^2+^ ions, thereby remodeling the brain microenvironment and enabling sensitive detection of early neuroinflammation prior to Ab plague formation in an APP transgenic TgCRND8+ AD mouse model.^2^ We also demonstrated its effects on improving vascular functions, Ab elimination, energy metabolism, neuronal activity and cognitive function.^3‐4^ Built on the foundation of previous findings, we investigate whether the TP‐MDNP is able enhance early detection of neuroinflammation regardless of Aß or tau expression and evaluate its therapeutic effect in AD mouse model of tauopathy.

**Method:**

Three types of transgenic mouse model of AD, TgCRND8+, PS19 with tauopathy, and APP/PS1 were used in the MRI study. The diagnostic performance of Ab‐TP‐MDNP in MRI was also compared with PET imaging using F18‐florobetaben in TgCRND8+ mice. PS19 mice were treated with IV injection of TP‐MDNP for two weeks (2/week, 100 μmol Mn/kg b.w.) and the biomarkers for ROS, neuroinflammation, and p‐Tau expression were examined using immunohistochemistry and ELISA.

**Result:**

Ab‐TP‐MDNP enhanced MRI signal significantly outperformed PET imaging by Ab‐targeted F18‐florobetaben in TgCRND8+ mice of 3 months and 6 months of age. Similar MRI imaging sensitivity was observed in PS19 and APP/PS1 mice with or without conjugated antibody against Ab or tau protein, suggesting the neuroinflammation activated MRI contrast enhancement as a common mechanism. In PS19, TP‐MDNP treatment significantly reduced total ROS, CA9 (a hypoxia marker) and p‐tau levels.

**Conclusion:**

The results suggest that TP‐MDNP can enable MRI detection of neuroinflammation and reduce neurodegeneration pathogenetic factors such as ROS, hypoxia, and p‐tau in AD mouse brains.

**References**

1. Zhang W, et al. *Sig Transduct Target Ther* 2023;8, 267.

2. He C, et al. *Nano Today*. 2020;35:100965.

3. Park E,. et al. *Advanced Science*. 2023 Apr;10(12):2207238.

4. Park E, et al. *Biomaterials*. 2025 Jan 24:123142.